# Recombinant oncolytic Newcastle disease virus displays antitumor activities in anaplastic thyroid cancer cells

**DOI:** 10.1186/s12885-018-4522-3

**Published:** 2018-07-18

**Authors:** Ke Jiang, Cuiping Song, Lingkai Kong, Lulu Hu, Guibin Lin, Tian Ye, Gang Yao, Yupeng Wang, Haibo Chen, Wei Cheng, Martin P. Barr, Quentin Liu, Guirong Zhang, Chan Ding, Songshu Meng

**Affiliations:** 1 Institute of Cancer Stem Cell, Dalian Medical University Cancer Center, Room 415, 9 Lvshun Road South, Dalian, 116044 China; 2 Department of Avian Infectious Diseases, Shanghai Veterinary Research Institute, Chinese Academy of Agricultural Sciences, 518 Ziyue Road, Shanghai, 200241 China; 3 Thoracic Oncology Research Group, Trinity Translational Medicine Institute, Trinity Centre for Health Sciences St. James’s Hospital and Trinity College Dublin, Dublin, Ireland; 4 Central laboratory, Liaoning Cancer Hospital and Institute, Cancer Hospital of China Medical University, 44 Xiaoheyan Road, Shenyang, 110042 China; 5 Laboratory Center, The Third People’s Hospital of Huizhou, Affiliated Hospital Guangzhou Medical University, Huizhou, 516002 China; 6 Department of Dermatology of First Affiliated Hospital, Dalian Medical University, No. 222 Zhongshan Road, Dalian, 116021 China

**Keywords:** Anaplastic thyroid cancer (ATC), Newcastle disease virus (NDV), p38 MAPK, Green fluorescent protein (GFP), Apoptosis

## Abstract

**Background:**

Anaplastic thyroid cancer (ATC) is one of the most aggressive of all solid tumors for which no effective therapies are currently available. Oncolytic Newcastle disease virus (NDV) has shown the potential to induce oncolytic cell death in a variety of cancer cells of diverse origins. However, whether oncolytic NDV displays antitumor effects in ATC remains to be investigated. We have previously shown that the oncolytic NDV strain FMW (NDV/FMW) induces oncolytic cell death in several cancer types. In the present study, we investigated the oncolytic effects of NDV/FMW in ATC.

**Methods:**

In this study, a recombinant NDV expressing green fluorescent protein (GFP) was generated using an NDV reverse genetics system. The resulting virus was named after rFMW/GFP and the GFP expression in infected cells was demonstrated by direct fluorescence and immunoblotting. Viral replication was evaluated by end-point dilution assay in DF-1 cell lines. Oncolytic effects were examined by biochemical and morphological experiments in cultural ATC cells and in mouse models.

**Results:**

rFMW/GFP replicated robustly in ATC cells as did its parent virus (NDV/FMW) while the expression of GFP protein was detected in lungs and spleen of mice intravenously injected with rFMW/GFP. We further showed that rFMW/GFP infection substantially increased early and late apoptosis in the ATC cell lines, THJ-16 T and THJ-29 T and increased caspase-3 processing and Poly (ADP-ribose) polymerase (PARP) cleavage in ATC cells as assessed by immunoblotting. In addition, rFMW/GFP induced lyses of spheroids derived from ATC cells in three-dimensional (3D) cultures. We further demonstrated that rFMW/GFP infection resulted in the activation of p38 MAPK signaling, but not Erk1/2 or JNK, in THJ-16 T and THJ-29 T cells. Notably, inhibition of p38 MAPK activity by SB203580 decreased rFMW/GFP-induced cleavage of caspase-3 and PARP in THJ-16 T and THJ-29 T cells. Finally, both rFMW/GFP and its parent virus inhibited tumor growth in mice bearing THJ-16 T derived tumors.

**Conclusion:**

Taken together, these data indicate that both the recombinant reporter virus rFMW/GFP and its parent virus NDV/FMW, display oncolytic activities in ATC cells in vitro and in vivo and suggest that oncolytic NDV may have potential as a novel therapeutic strategy for ATC.

**Electronic supplementary material:**

The online version of this article (10.1186/s12885-018-4522-3) contains supplementary material, which is available to authorized users.

## Background

Anaplastic thyroid cancer (ATC) is the most aggressive type among thyroid cancers, accounting for a significant portion of thyroid cancer death [1]. Current treatments for ATC patients such as surgery, radiotherapy and chemotherapy have no effect in increasing patients’ survival [2]. Therefore, the development of novel therapeutic approaches for ATC is urgently needed.

Oncolytic viruses (OVs) are naturally occurring or engineered viruses that selectively infect and replicate in cancer cells, triggering direct oncolysis. Several preclinical studies have demonstrated that OV-based therapy is effective in the treatment of ATC [3]. A series of studies by Portella & colleagues has shown that oncolytic adenovirus strains dl1520 (Onyx-015) and dl922–947, alone or in combination with rationally designed molecularly-targeted drugs, displayed antitumor activities in ATC cells and in in vivo mouse models [4–9]. Similarly, the adenovirus strain, ONYX-411, induced cell death in ATC cell lines and suppressed the growth of xenograft tumors in nude mice [10]. In addition to oncolytic adenoviruses, oncolytic vaccina viruses also displayed antitumor activities in ATC cells and in xenograft models [11, 12]. Wong et al. investigated the oncolytic effects of oncolytic vaccina virus strains NV1023 and GLV-1 h68 in ATC in the preclinical setting [13–16]. Other OVs such as measles virus has also been demonstrated to induce cytotoxicity in ATC cells [17]. Together, these studies strongly indicate that OVs hold promise for the treatment of patients with ATC.

Newcastle disease virus (NDV) is a member of the Avulavirus genus in the Paramyxoviridae family. Naturally occurring strains of NDV and recombinant NDV expressing immunoregulatory factors have demonstrated the potential to kill cancer cells of diverse origin in both preclinical and clinical studies [18, 19]. However whether oncolytic NDV displays antitumor effects in ATC remains to be investigated. We have previously shown that either naturally occurring or recombinant oncolytic NDV expressing apoptin triggers oncolytic cell death in lung and liver tumor cell lines and tumor-bearing mice [20–24]. The aim of the present study was to determine the oncolytic efficacy of NDV using a recombinant NDV-expressing GFP protein in ATC cell lines and mouse model. To better understand oncolytic NDV infection process in cancer cells, we generated a recombinant NDV expressing the green fluorescent protein (GFP). We evaluated the efficacy of the recombinant NDV in ATC cell lines and in mouse models. Our results show that the GFP-expressing reporter NDV, exhibits potent oncolytic activities in ATC cell lines and in a mouse model of thyroid cancer.

## Methods

### Cells, viruses and regent

Chicken embryo fibroblast cell line, DF1 (cat no. GNO30), was obtained and authenticated by the Cell Bank of the Chinese Academy of Science (Shanghai, China). Cells were maintained in DMEM supplemented with 10% fetal bovine serum (FBS). THJ-16 T and THJ-29 T cells were kindly provided by the Mayo Foundation for Medical Education and Research to Dr. Quentin Liu [25]. THJ-16 T cells were cultured in RPMI-1640 containing 5% FBS, 10 mM HEPES (Thermo Fisher) and 1 mM sodium pyruvate (Thermo Fisher). THJ-29 T cells were cultured in RPMI-1640 containing 5% FBS and 1 mM sodium pyruvate. A virulent strain of NDV/FMW (GenBank accession number: GU564399) was prepared as reported previously [20]. SB203580, a specific p38 inhibitor, was purchased from Selleckchem which was prepared with dimethyl sulfoxide (DMSO) and stored at − 20°C.

### Construction of GFP-labelled recombinant NDV/FMW

The construction of the recombinant NDV/FMW expressing GFP was performed essentially as described in our previous study for the generation of the recombinant NDV/FMW expressing apoptin [24]. To construct rFMW-GFP, a GFP-labeled fragment flanked by the appropriate NDV-specific RNA transcriptional signals was inserted into the ApaI site created between the P and M genes of pT7NDV/FMW. The resulted plasmid was named as rFMW-GFP and sequencing verified. Viruses were rescued from complementary cDNA using methods described previously [24]. The resultant recombinant virus, rFMW/GFP was prepared, stored and titered as previously described [20–22, 24].

### Live cell imaging

THJ-16 T and THJ-29 T cells were cultured in 6-well plates and infected with NDV/FMW or rFMW/GFP at a multiplicity of infection (MOI) of 10. Cells were observed using fluorescence microscopy (Olympus IX81). Live cell imaging of bright-field and fluorescence was recorded at 24 h post-infection.

### Immunofluorescence assay

THJ-16 T and THJ-29 T cells for immunofluorescence assay were seeded on coverslips (NEST, 801008) in 24 wells plate and fixed in 4% paraformaldehyde (PFA) for 30 min, then the cells were permeabilized in 0.2% Triton X-100 for 15 min. Non-specific binding sites were blocked by incubation with 3% Bovine Serum Albumin (BSA) for 60 min. Cells were then incubated with primary anti-HN antibody (1:50) overnight at 4 °C. After washed, secondary antibodies (1:1000) were added to appropriate wells. After 60 min, Nuclei were stained with DAPI (5 μg/mL, Sigma) in PBS. Images were acquired using a confocal microscope (Leica TCS SP5 ×) and images were captured with a camera controlled. Images from each experiment were acquired using the same exposure time during the same imaging session.

### Immunoblot assay

THJ-16 T and THJ-29 T cells were seeded in 60-mm dishes and infected with vehicle or rFMW/GFP at 10 MOI. Cells were harvested using scraper and lysed in lysis buffer (Roche, USA) at 6, 12 or 24 h. Cell samples were loaded and separated by 10 or 15% SDS-PAGE and subsequently transferred to nitrocellulose membranes (Applygen Technologies Inc. Beijing, China) using a transblot turbo system. Membrane was blocked with 5% milk diluted in TBST buffer (0.05% Tween-20) for 3 h and incubated with primary antibody at 4 °C overnight. The antibodies for GFP-tag (1:10000, Sigma, SAB4301138), HN (1:500, Santa cruz, SC-53562), β-actin (1:10000, Sigma, A1978), caspase-3 (1:1000, Cell signaling technology, 9662S), PARP (1:1000, Cell signaling technology, 9532S), phospho-p38 (1:1000, Cell signaling technology, 9215S), total p38 (1:1000, cell signaling technology, 9212S), phospho-Erk1/2 (1:8000, Promega, V803A), total Erk1/2 (1:8000, Promega, V114A), phospho-JNK (1:2000, Cell signaling technology, 9251S) and total JNK (1:1000, Cell signaling technology, 9253S) were used. After washed three times with TBST, the membranes were incubated with horseradish peroxidase-conjugated secondary antibody (1:10000, Invitrogen, USA) for 1 h at room temperature with continuous rocking. The blots were detected using ECL Western Blot Substrate kit (Thermo Fisher, USA) [20].

### GFP expression in vivo

rFMW/GFP (1 × 10^7^ TCID50 per dose) was injected intravenously (i.v) into BALB/c mice. To assess GFP expression in organs, mice were euthanized 24 h following virus injection. Heart, liver, spleen, lungs and kidneys were harvested. Cells were lysed and GFP protein was visualized by IB.

### Viral titer assay

DF1 cells were seeded in 96-well plates and then infected with 10-fold serially diluted viruses. Viral titer was measured by end-point dilution assay (50% tissue culture infective dose (TCID50]/ml) and the TCID50 was calculated by the method of Reed and Muench (Reed and Muench, 1938).

### Cell viability assay

Cell viability was quantified using the 3-(4,5-dimethylthiazol-2-yl)-2,5-diphenyltetrazolium bromide (MTT) assay based on the formation of formazan crystals from tetrazolium by living/metabolically active cells. THJ-16 T and THJ-29 T cells were seeded in 96-wells plates (5000 cells/well), and then the cells were vehicle-infected or infected with varying MOI of rFMW/GFP (0.01, 0.1, 1, and 10) for 24, 48, 72 h. Cell growth inhibition was determined as previously described [22].

### Spheroid formation

THJ-16 T and THJ-29 T spheroids were prepared from monolayer cells which were trypsinised and plated in ultra-low attachment 96-well plates (1000 cells/well). The cells were containing in serum-free DMEM/F12 medium supplemented with 10 ng/ml basic fibroblast growth factor (bFGF), 20 ng/ml epidermal growth factor (EGF) and 1 × B27. After 7 days, the propagated spheroid bodies were observed and counted by light microscope.

### In vivo oncolysis

Female age-matched (6 weeks old) nude mice were housed in specific pathogen-free (SPF) conditions. THJ-16 T cell suspension (5 × 10^6^ cells in 100 μL PBS/mouse) was injected subcutaneously in the right flank to induce tumor development. When tumors reached an average volume of 200 mm^3^, the rFMW/GFP treatments were initiated by intratumoral injection. Mice were randomly divided into two groups (eight mice per group): (a) vehicle control, (b) intratumoral administration with rFMW/GFP (1 × 10^7^ TCID50 per dose). Mice were injected three times weekly. After 1 week, four mice (of eight) were euthanized to make into slices. The slices were subjected to either hematoxylin-eosin (H&E) staining or terminal deoxynucleotidyl transferase dUTP nick end labeling (TUNEL) assay as previously described [24]. The total proteins of tumor tissue samples were harvested from the other four mice in each group to test GFP and HN expression by immunoblot assay.

For the in vivo oncolysis study of growth curve, ten mice were included in each group ((a) vehicle control, (b) intratumoral administration with rFMW/GFP (1 × 10^7^ TCID50 per dose), (c) intratumoral administration with NDV/FMW (1 × 10^7^ TCID50 per dose), was treated for 3 weeks. Tumor growth was monitored at 5-day intervals for 50 days using and volume was determined with digital caliper according to the formula: volume = (greatest diameter) × (smallest diameter)^2^/2. Euthanasia: Treat mice in an inhalation anesthesia machine (Shanghai Biowill Co., LTD, Model: BW-AM503) with 5% isoflurane (Sigma, cat no. Y0000858); 2.4 L/min N2O; 1.2 L/min O2. Observe the mice. (The depth of anesthetization is sufficient when the following vital criteria are reached: regular spontaneous breathing. No reflex after setting of pain stimuli between toes, and no response to pain.) Carotid after anesthesia mice was killed off. The animals were tested in a biosafety cabinet of the SPF laboratory animal center of the Dalian Medical University (Dalian, China), complying with the national guidelines for the care and use of laboratory animals and were approved by the experimental animal ethics committee at Dalian Medical University.

### Statistical analysis

For all experiments, statistical analysis was first performed using a one-way analysis of variance (ANOVA) to determine statistical significance between groups for each endpoint assessed. Multiple comparisons between treatment groups and controls were evaluated using Dunnett’s LSD test. To assess the in vivo oncolytic effects, statistical significance between groups was calculated using the LSD post-test and SPSS 11.0 software (SPSS Inc., Chicago, IL, USA). *p*-values< 0.05 was considered statistically significant.

## Results

### Construction of recombinant NDV expressing GFP

**Fig. 1:**
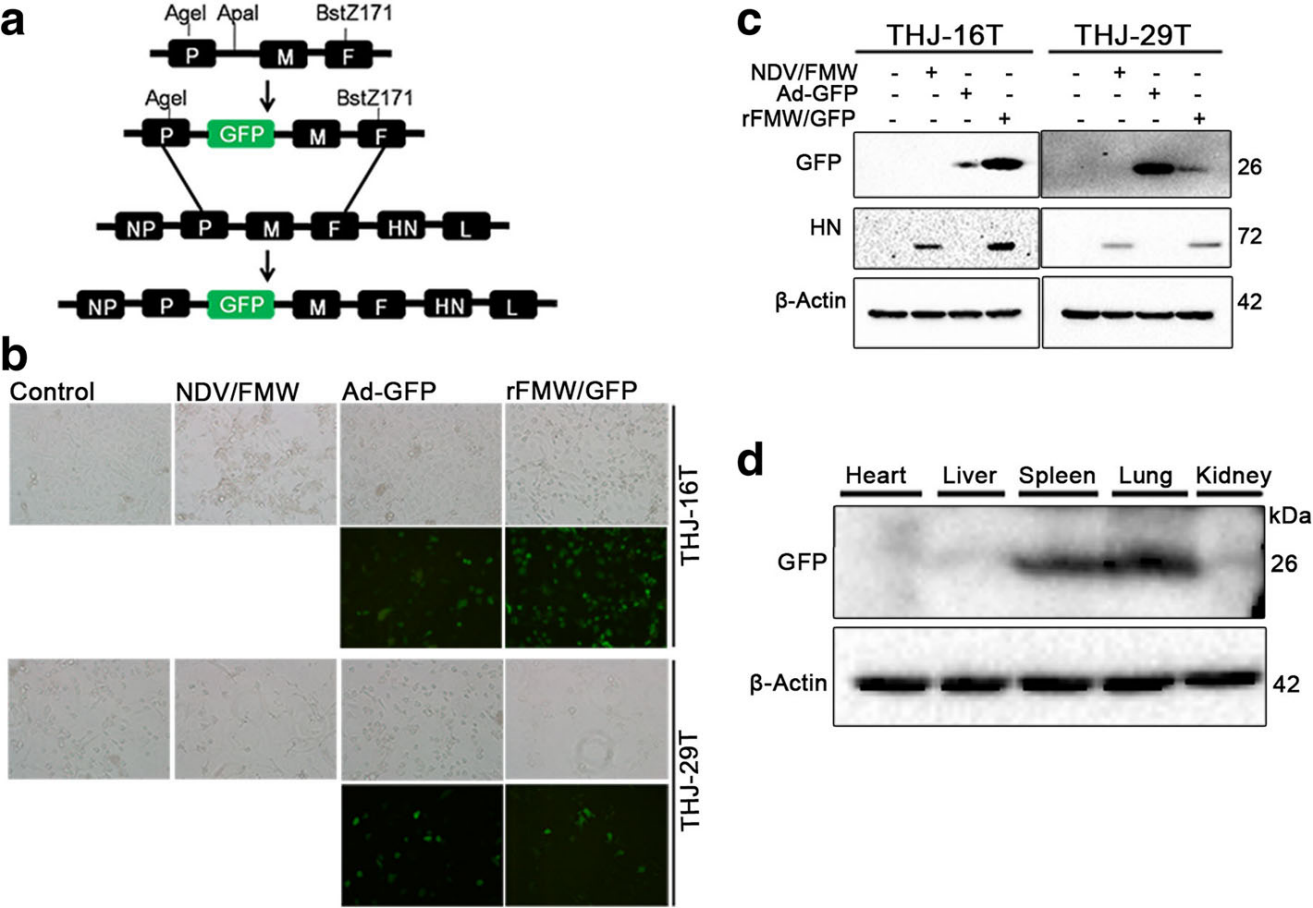
Construction and identification of the recombinant rFMW/GFP. **a** PCR amplification of the DNA construct using ApaI-tagged primers was carried out and subsequently introduced into the Anti-sense cDNA of the NDV strain, FMW. **b** THJ-16 T and THJ-29 T cells were infected with vehicle, NDV/FMW (10 MOI), adenovirus-GFP-Tag or rFMW/GFP (10 MOI) and imaged by fluorescence microscopy. Live cell imaging using bright-field and fluorescence microscopy was recorded at 24 h post-infection. **c** Protein levels of GFP-tagged and HN proteins were analyzed by immunoblotting (IB). **d** rFMW/GFP (1 × 10^7^ TCID50 per dose) was i.v. injected into BALB/c mice. Mice were sacrificed 24 h post virus injection. Heart, liver, spleen, lungs and kidneys were harvested and GFP protein expression was assessed by IB. β-actin was used as a control for equal loading. All IB experiments were performed twice

We have previously shown that the NDV strain FMW (NDV/FMW) exhibits strong oncolytic activity in vitro and in vivo [20–23]. To better monitor the NDV/FMW infection process in cancer cells, we generated recombinant NDV carrying the GFP reporter gene (Fig. 1a). The construction of the recombinant NDV/FMW expressing GFP (hereafter as rFMW/GFP) was performed utilizing the reverse genetics rescue system, which was employed in our previous study for the generation of the recombinant NDV/FMW expressing apoptin [24].

GFP expression in rFMW/GFP-infected ATC cells (THJ-16 T and THJ-29 T) was demonstrated by direct fluorescence and immunoblotting (Fig. 1b and c). The expression of HN protein was detected in NDV/FMW-infected cells (Fig. 1c). Importantly, detection of GFP was accompanied by the expression of HN protein in rFMW/GFP-infected cells (Fig. 1c), indicating the replication of rFMW/GFP in infected cells. In addition, the stability of the recombinant virus was evaluated by inoculating rFMW/GFP into SPF embryonated chicken eggs after five serial passages. GFP insertion was subsequently confirmed by RT-PCR assay (data not shown). To determine the in vivo distribution of rFMW/GFP, expression of GFP protein in organs from mice intravenously injected with rFMW/GFP was analyzed by immunoblotting. GFP protein was detected in spleen and lung (Fig. 1d), indicating the tissue tropism of rFMW/GFP and demonstrates the successful construction of a recombinant oncolytic NDV expressing GFP.

### Replication characteristics of rFMW/GFP

**Fig. 2:**
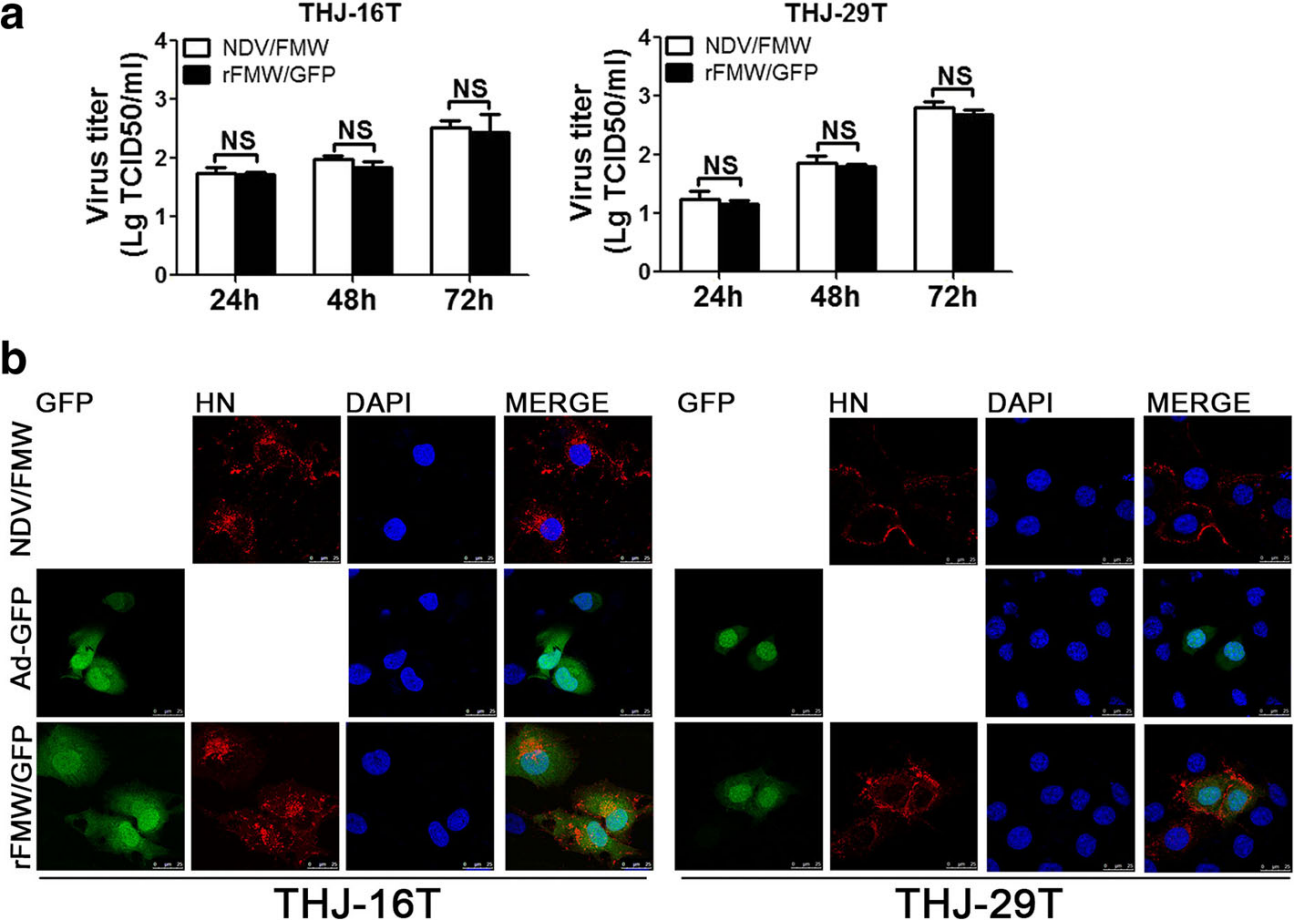
Replication characteristics of rFMW/GFP. **a** THJ-16 T and THJ-29 T cells were infected with NDV/FMW (0.01 MOI) or rFMW/GFP (0.01 MOI) for 24, 48 and 72 h respectively. Virus yield was determined at different intervals. Each assay was repeated three times. Data are presented as the mean ± SD for three independent experiments. **b** THJ-16 T and THJ-29 T cells were infected with NDV/FMW (10 MOI), Adenovirus-GFP-Tag or rFMW/GFP (10 MOI) for 24 h, stained with rabbit anti-HN polyclonal antibody and visualized by confocal microscopy using three channels (405, 488 and 643 nm). DAPI was used for nuclear staining

To investigate whether GFP insertion caused any replication defect in NDV/FMW, the growth characteristics of rFMW/GFP and its parent virus were evaluated in a single-step growth cycle in DF-1 cell lines. The insertion of GFP into the NDV/FMW genome did not significantly influence viral replication kinetics and total viral yield during the 72 h post-infection (hpi) period compared to the parental NDV/FMW strain (Fig. 2a). However, detection of GFP was accompanied by the expression of HN protein in rFMW/GFP-infected THJ-16 T and THJ-29 T cells by confocal microscopy (Fig. 2b), indicating the successful replication of rFMW/GFP in the ATC cell lines tested.

### Oncolytic activity of rFMW/GFP in ATC cell lines using 2D and 3D cultures

**Fig. 3:**
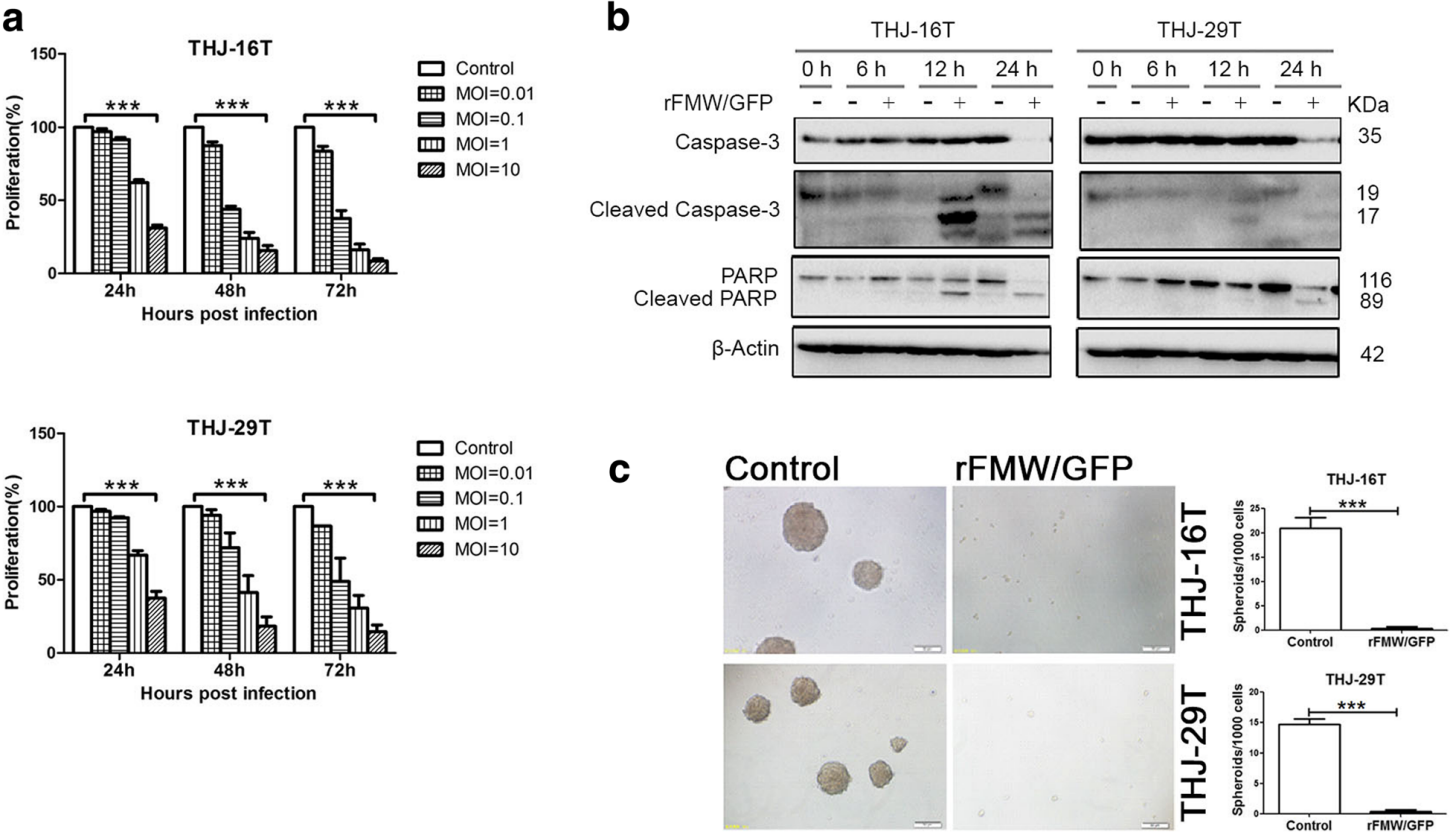
Oncolytic activity exhibited by rFMW/GFP against ATC cells in both 2D and 3D cultures. **a** THJ-16 T and THJ-29 T cells were vehicle-infected or infected with varying MOI of rFMW/GFP (0.01, 0.1, 1 and 10) for 24, 48, 72 h respectively. Cell growth inhibition was determined using the MTT assay. **b** THJ-16 T and THJ-29 T cells were infected (similar to A above) with the same as in A for 6, 12 and 24 h. Expression levels of total and cleaved caspase-3 and PARP were analyzed by IB. β-actin was used as a control for equal loading. **c** 3D cultures of THJ-16 T and THJ-29 T cells were infected (similar to A above) for 48 h and examined for spheroid formation. Results are expressed as number of spheroids/1000 cells ± SEM, ***p < 0.001. (Scale bar = 100 μm). Data are representative of the mean ± SEM (***p < 0.001)

We proceeded to determine whether rFMW/GFP induced growth inhibition in ATC cell lines. rFMW/GFP infection induced a significant reduction in viability of THJ-16 T and THJ-29 T cell lines in a time and concentration-dependent manner compared to mock infections (Fig. 3a). Similar results were obtained in parent NDV/FMW-infected THJ-16 T and THJ-29 T cells (data not shown). To examine whether the rFMW/GFP-induced growth inhibition of ATC cells was due to apoptosis, caspase-3 and Poly (ADP-ribose) polymerase (PARP) cleavage, two classical markers of apoptosis, was demonstrated in rFMW/GFP-treated THJ-16 T and THJ-29 T cells at 12 hpi as assessed by immunoblotting (Fig. 3b). Together, these data indicated that rFMW/GFP induced apoptosis in ATC cells.

Given that tumor spheroids are considered a useful in vitro model to mimic the biological properties of tumors, we examined whether the effects of rFMW/GFP on spheroids derived from ATC cells in a three-dimensional (3D) culture system. Upon rFMW/GFP infection at an MOI of 10 for 48 h, rFMW/GFP-infected THJ-16 T or THJ-29 T cells did not grow under these conditions compared to mock-infected cells (Fig. 3c).

### Regulating p38 MAPK signaling by rFMW/GFP

**Fig. 4:**
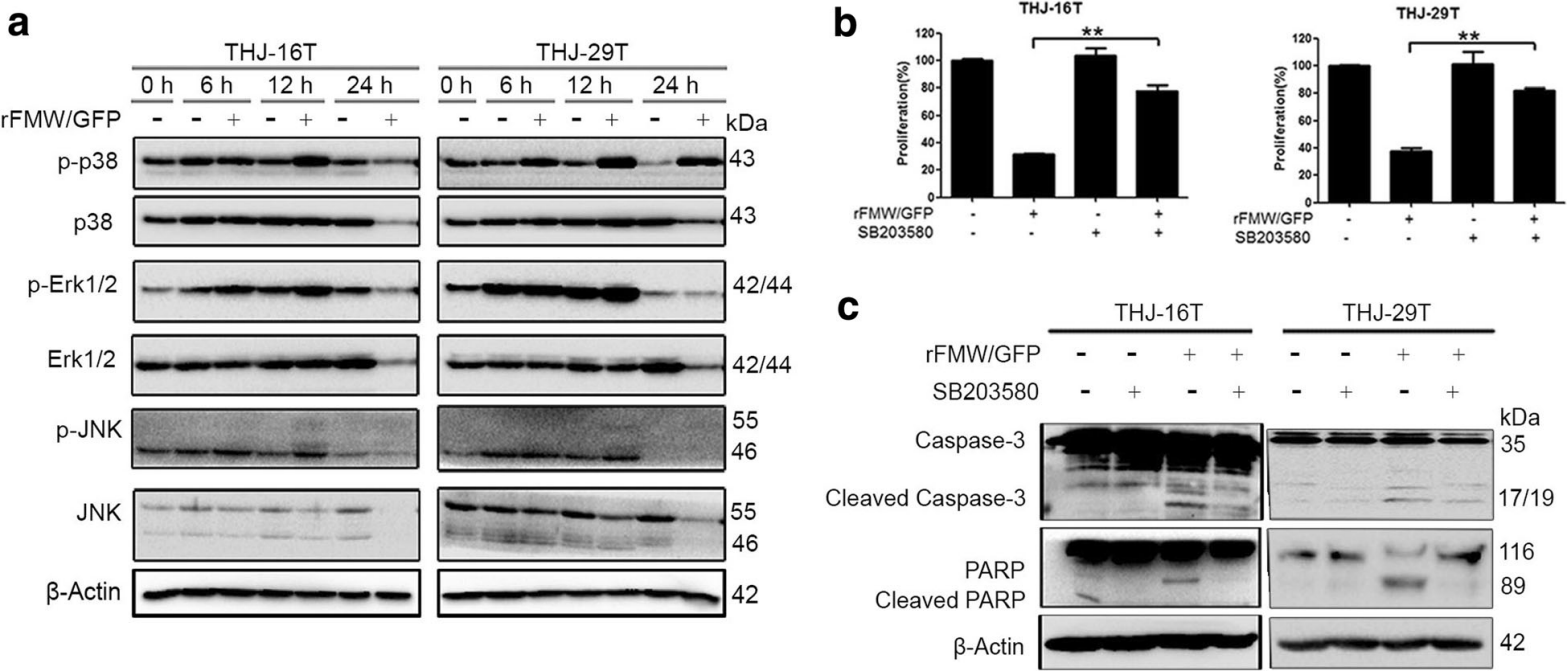
Signaling pathways targeted by rFMW/GFP in ATC cell lines. **a** THJ-16 T and THJ-29 T cells were infected with rFMW/GFP for 6, 12 and 24 h. Protein levels of p-p38, total p38, p-Erk1/2, total Erk1/2, p-JNK and total JNK were analyzed by immunoblotting (IB). β-actin was used as a control for equal loading. **b** THJ-16 T and THJ-29 T cells were infected with vehicle or 10 MOI rFMW/GFP following pre-treatment with the p38 MAPK inhibitor, SB203580 (10 μM). Cell growth inhibition was determined using the MTT assay. Data are presented as the mean ± SEM, 0.001 < **p < 0.005. **c** Protein expression of total and cleaved caspase-3 and PARP was examined by IB

We sought to explore the potential signaling pathways involved in rFMW/GFP-triggered oncolytic cell death in ATC cells. We examined the role of the MAPK pathway (Erk1/2, JNK and p38) as potential downstream signaling MAPK proteins, as we had previously shown these to be implicated in NDV/FMW-induced cytotoxic effects on lung cancer cells [20, 21]. p38 MAPK signaling was activated in both THJ-16 T and THJ-29 T cells upon rFMW/GFP infection at 12 hpi and in THJ-29 T cells at 24 hpi. While phosphorylation of either Erk1/2 or JNK was not significantly altered in either cell line (Fig. 4a). In addition, we examined other critical pathways such as Akt and p53 signaling which are generally involved in cell survival and apoptosis in THJ-16 T and THJ-29 T cells infected with rFMW/GFP. As shown in the Additional file 1: Figure S1, total AKT level and p53 level were all downregulated after a 24-h infection with rFMW/GFP, suggesting that both Akt and p53 signaling might play a role in the antitumor effects by rFMW/GFP. But change in p-Akt (S473) was not consistent between the two cell lines upon infection with rFMW/GFP (Additional file 1: Figure S1). To investigate the role of p38 MAPK pathway in rFMW/GFP-induced cell death in ATC cells, the specific p38 MAPK inhibitor, SB203580, was added to THJ-16 T and THJ-29 T cells 30 min prior to virus infection. Treatment with SB203580 significantly reduced rFMW/GFP-induced cell death in both cell lines at 24 hpi. Compared to mock-infected controls (virus only) (Fig. 4b). Inhibition of p38 MAPK activity by SB203580 decreased rFMW/GFP-induced cleavage of caspase-3 and PARP in THJ-16 T and THJ-29 T cells (Fig. 4c). These data suggest a role for p38 MAPK in rFMW/GFP-induced oncolysis of ATC cells.

### Antitumor effects of rFMW/GFP in mice bearing 26 T-deprived tumors

**Fig. 5:**
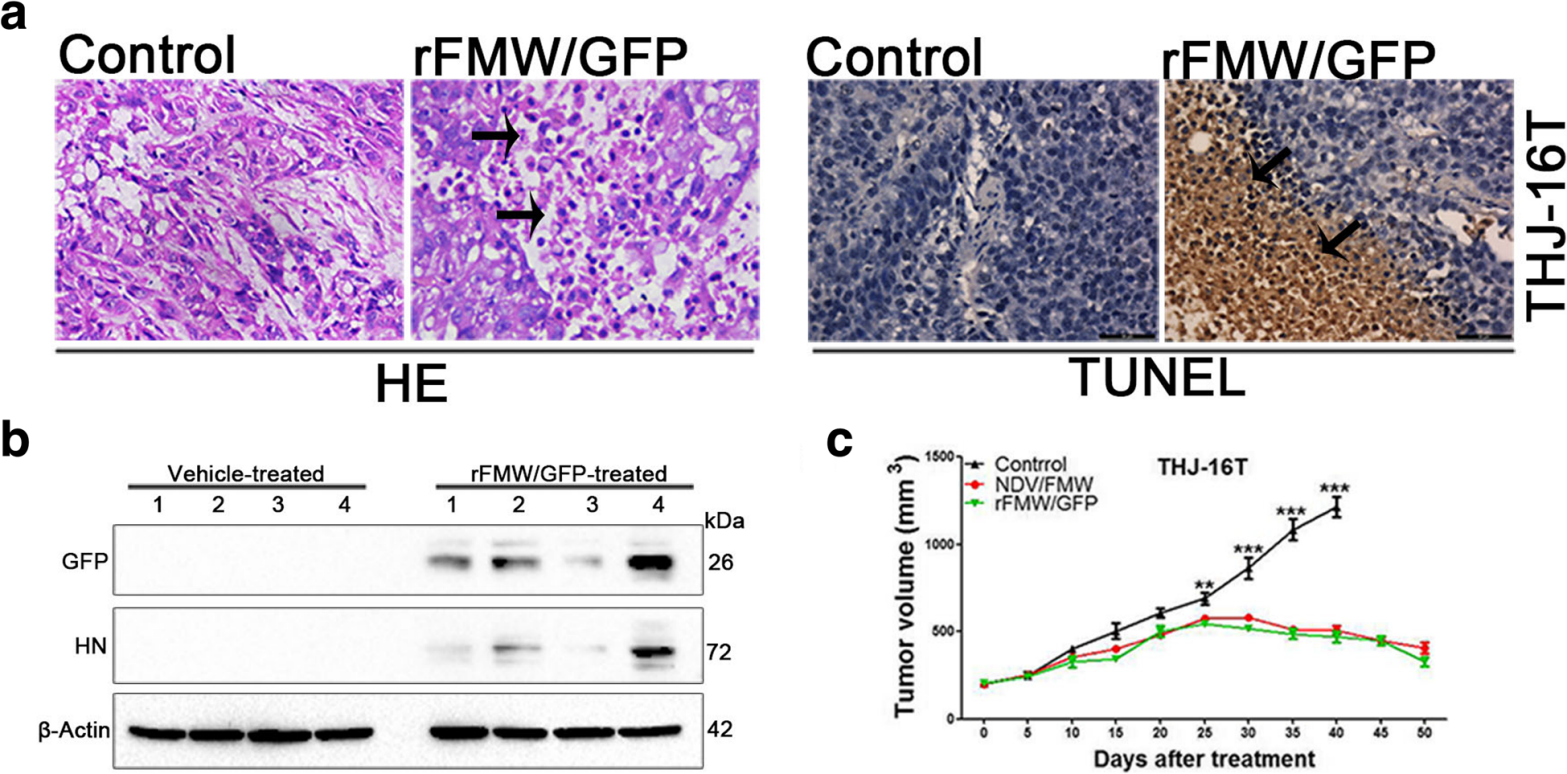
In vivo antitumor effects of rFMW/GFP. **a** One week after treatment, tumor tissue samples from four different animals from each treatment group (of eight) were subjected to either hematoxylin-eosin (H&E) staining (Tumor necrosis indicated by the arrows) or TUNEL assay (Arrowheads indicate brown 3,3′-diaminobenzidine chromogen in cell nuclei) or **b** immunoblot analysis of GFP and HN expression. β-actin was used as a loading control. Scale bar = 50 μm. **c** Mice were treated as described above for 3 weeks. Tumor volumes were measured at 5-day intervals for 50 days after injections and expressed as the mean ± SEM (*n* = 10) in tumor volume-time curves. Differences in tumor regression were significant between virus-treated and vehicle control groups. Data are expressed as mean ± SEM and are representative of two independent experiments (0.001 < **p < 0.005; ***p < 0.0001)

The oncolytic effects of rFMW/GFP and its parent virus NDV/FMW were investigated in mice bearing tumors derived from THJ-16 T cells. The design of these in vivo experiments were based on previous studies from our lab and others [21, 22, 24, 26, 27]. Tumor sections were examined by H&E staining and TUNEL assay. H&E staining of tumors treated with rFMW/GFP showed characteristic apoptotic cells (Fig. 5a). In addition, the TUNEL assay revealed pyknotic chromatin in virus-inoculated tumors (Fig. 5a). In contrast, fewer necrotic and apoptotic cells were detected in PBS-treated controls. Interestingly, immunoblotting analysis of tumor lysates demonstrated GFP and HN expression in rFMW/GFP-inoculated tumors but not in PBS-treated tumors (Fig. 5b). Moreover, viruses isolated from tumors infected with rFMW/GFP, was shown to be infectious (data not shown), indicating that rFMW/GFP replicated in virus-treated tumors.

Consistent with these in vivo data, significant tumor regression was observed in mice inoculated with either rFMW/GFP or parent virus compared to control groups (Fig. 5c). However, there was no significant difference in tumor growth inhibition between rFMW/GFP and parent virus. Non-tumor-bearing mice injected with either rFMW/GFP or parent virus survived and remained healthy during the course of this in vivo study.

## Discussion

Although oncolytic NDV is emerging as a novel cancer therapeutic approach in the treatment of a variety of cancer types, including thyroid cancer [28], only one early report by Zamarin et al. showed NDV as an effective oncolytic agent against thyroid cancer cell lines in an in vitro study [18]. In addition, no clinical trial has been initiated with oncolytic NDV for thyroid cancer. Therefore, to our knowledge; this is the first report demonstrating that oncolytic NDV targets ATC in vitro and in vivo. We showed that the NDV/FMW strain and its derived recombinant expressing GFP, rFMW/GFP, induced cytotoxicity in ATC cells in both 2D and 3D cultures and in mice bearing ATC cell-derived tumors. Thus, our study suggests the use of oncolytic NDV as a promising therapeutic strategy for ATC.

To better track oncolytic NDV in vitro and in vivo, several oncolytic NDV strains such as D90, F3aa and Italien, have been engineered to express GFP [26, 27, 29–31]. In our previous study, the oncolytic NDV strain FMW was used as a vector to express apoptin to enhance the effects of NDV/FMW in cancer cells [24]. In the present study, the GFP gene was inserted into the genome of NDV/FMW and the resultant virus, rFMW/GFP, replicated robustly in ATC cells as did its parent virus. Furthermore, GFP expression was observed in rFMW/GFP-infected ATC cell lines and in tumor sections from mice bearing ATC cell-derived tumors, indicating that rFMW/GFP can be used as a reporter virus to probe the infection process in vitro and in vivo. Moreover, analysis of the distribution of rFMW/GFP indicated that expression of GFP protein was detected in lung and spleen of mice intravenously injected with rFMW/GFP, in line with a previous study by Bian et al. in mice intravenously injected with the recombinant NDV strain, NDFL-EGFP [32]. Interestingly, in non-human primates, intravenous injection with oncolytic NDV resulted in the accumulation of the viral RNA in the respiratory tract, spleen and liver [33]. Together, our data add further knowledge to the current understanding of the preclinical efficacy of rFMW/GFP in thyroid cancer cells, in addition to the administration of oncolytic NDV in animal models.

Our previous studies have shown that NDV/FMW induces apoptosis in a variety of cancer cells, during which the MAPK pathways were disturbed [20–23]. Analysis of the signaling pathway involved in rFMW/GFP-induced apoptosis revealed that p38MAPK, but not Erk1/2 or JNK, was activated in infected ATC cells. Furthermore, inactivation of p38MAPK activity attenuated the cytotoxic effects of rFMW/GFP on ATC cells, supporting a role of p38 MAPK in rFMW/GFP-induced oncolytic activity in thyroid cancer cells. These data together with our previous observations that p38 MAPK plays a role in NDV/FMW-triggered apoptosis in lung cancer cells [20, 21], highlight that p38 MAPK plays a role in the induction of apoptosis by oncolytic NDV in a variety of cancer types.

In summary, we present evidence showing that both the recombinant reporter virus rFMW/GFP and its parent virus NDV/FMW display oncolytic activities in ATC cells in vitro and in vivo. Furthermore, rFMW/GFP will be an important tool for tracing the efficacy of NDV/FMW in target cancer cells and for further elucidating the mechanism(s) by which NDV/FMW induces oncolytic cell death.

## Conclusions

In the present study, we identified recombinant reporter virus rFMW/GFP display oncolytic activities in ATC cells via p38 MAPK signaling pathway and represent a novel potential therapeutic strategy for ATC.

## Additional file

Additional file 1: **Figure S1.** Akt and p53 signaling in the antitumor effects by rFMW/GFP. THJ-16 T and THJ-29 T cells were infected with rFMW/GFP for 6, 12 and 24 h. Protein levels of p-Akt (S473), total Akt and p-53 were analyzed by immunoblotting (IB). β-Actin was used as a control for equal loading. (JPG 100 kb)

## Abbreviations

3D: Three-dimensional
ATC: Anaplastic thyroid cancer
bFGF: Basic fibroblast growth factor
BSA: Bovine Serum Albumin
EGF: Epidermal growth factor
FBS: Fetal bovine serum
GFP: Green fluorescent protein
MTT: 3-(4, 5-dimethylthiazol-2-yl)-2, 5-diphenyltetrazolium bromide
NDV: Newcastle disease virus
OV: Oncolytic virus
PFA: Paraformaldehyde
