# Supplementary figures and images for: Recombinant oncolytic Newcastle disease virus displays antitumor activities in anaplastic thyroid cancer cells
Source: BMC Cancer. 2018 Jul 18;18:746. doi: 10.1186/s12885-018-4522-3 (PMC6052588; doi:10.1186/s12885-018-4522-3)

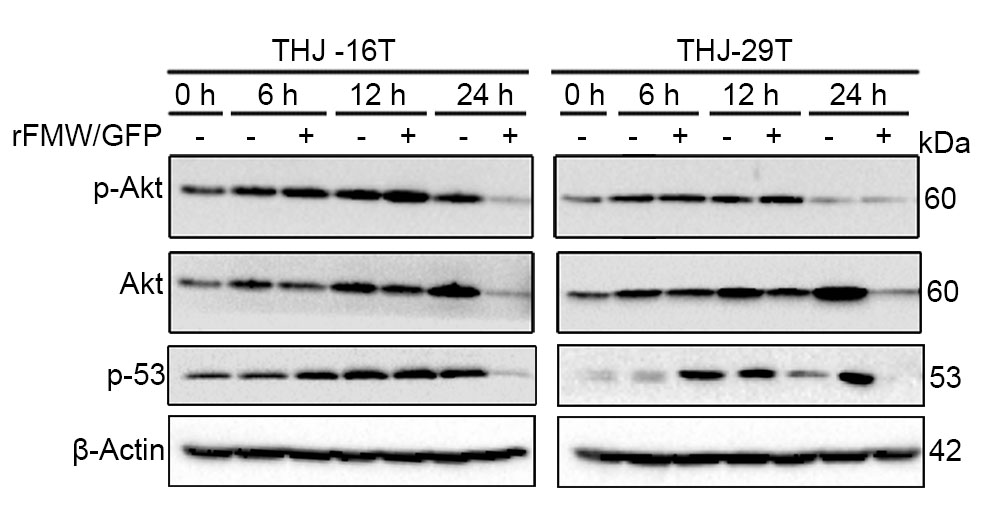

Supplement: Supplementary file 1 — Figure S1. Akt and p53 signaling in the antitumor effects by rFMW/GFP. THJ-16 T and THJ-29 T cells were infected with rFMW/GFP for 6, 12 and 24 h. Protein levels of p-Akt (S473), total Akt and p-53 were analyzed by immunoblotting (IB). β-Actin was used as a control for equal loading. (JPG 100 kb) [file 12885_2018_4522_MOESM1_ESM.jpg]
